# Direct regulation of FOXK1 by C-jun promotes proliferation, invasion and metastasis in gastric cancer cells

**DOI:** 10.1038/cddis.2016.225

**Published:** 2016-11-24

**Authors:** Ying Peng, Pei Zhang, Xiaoting Huang, Qingqing Yan, Meiyan Wu, Ruyi Xie, Yao Wu, Mengnan Zhang, Qingzhen Nan, Jinjun Zhao, Aimin Li, Jing Xiong, Yuexin Ren, Yang Bai, Ye Chen, Side Liu, Jide Wang

**Affiliations:** 1 Guangdong Provincial Key Laboratory of Gastroenterology, Department of Gastroenterology, Nanfang Hospital, Southern Medical University, Guangzhou 510515, China; 2 Department of Gastroenterology, Guangzhou First People's Hospital, Guangzhou Medical University, Guangzhou 510180, China; 3 Department of Gastroenterology, Guangzhou Nansha Central Hospital, Guangzhou 511457, China; 4 Department of Rheumatism, Nanfang Hospital, Southern Medical University, Guangzhou 510515, China

## Abstract

Forkhead box (FOX) K1 is a member of the FOX transcription factor superfamily. High FOXK1 expression is associated with several cancers. However, whether FOXK1 expression contributes to gastric cancer (GC) development and progression remains unknown. We analyzed the FOXK1 promoter using the Promo software and found several binding sequence transcription factors, including c-jun. However, the molecular mechanism by which FOXK1 affects the c-jun-mediated malignant phenotype is poorly understood. Here, we found that FOXK1 protein expression was higher in 8/10 (80.0%) fresh cancer tissues compared with that in adjacent normal tissues. FOXK1 overexpression enhanced the proliferation, migration and invasion of GC cells. Moreover, FOXK1 expression was stimulated by transforming growth factor-*β*1 (TGF-*β*1). FOXK1 acted as a potential epithelial-to-mesenchymal transition (EMT) inducer by stimulating vimentin expression and inducing the loss of E-cadherin in stable FOXK1-transfected cells. The results of promoter reporter and chromatin immunoprecipitation assays demonstrated that c-jun directly binds to and activates the human FOXK1 gene promoter. A positive correlation was observed between the expression patterns of FOXK1 and c-jun in GC cells and tissue. FOXK1 and c-jun expression were correlated with tumor progression and represented significant predictors of overall survival in GC patients. However, the siRNA-mediated repression of c-jun in FOXK1-overexpressing cells reversed EMT, as well as the proliferative and metastatic phenotypes. *In vivo*, c-jun promoted FOXK1-mediated proliferation and metastasis via orthotopic implantation. The evidence presented here suggests that FOXK1-directed regulation by c-jun promote the development and progression of human GC.

The *Drosophila* transcription factor forkhead and subsequent mammalian orthologs of the forkhead DNA-binding domain were discovered over two decades ago.^1^ Forkhead transcription factors encode a subgroup of helix–turn–helix proteins.^2^ The arrangement of loops connecting the *β*-strands that flank one of the three *α*-helices gives rise to a butterfly-like appearance (hence, these proteins are termed 'winged-helix' transcription factors).^3^ Through the transcriptional control of gene expression, many FOX protein members have important roles in embryonic development,^4^ organogenesis and regulation of a variety of physiological processes, such as cell cycle progression,^5^ cell survival,^6^ cellular metabolism,^7^ lifespan control^8^ and immune responses.^9^ Consequently, dysregulation of the functions, subcellular localization and expression of FOX transcription factors leads to the development and progression of diseases, especially cancer.^10, 11, 12^ For example, FoxQ1 induces the epithelial-to-mesenchymal transition (EMT) through transactivating ZEB2 expression by directly binding to the ZEB2 promoter.^13^ ZEB2 knockdown decreases FoxQ1-enhanced HCC metastasis, whereas ZEB2 upregulation rescued the decreased metastasis induced by knockdown of FoxQ1.

The human *FOXK1* gene encodes predicted proteins that are most homologous to the mouse myocyte nuclear factor (MNF)/Forkhead box K1 (Foxk1).^14^ Murine FOXK1 (Foxk1/MNF) exists as two isoforms, MNFa and MNFb (the C terminally truncated MNFb isoform is produced through alternative splicing).^15^ For the human *FOXK1* gene, protein feature analysis predicted the existence of a forkhead domain, an FHA domain and a nuclear localization sequence.^16^ Yang *et al.*^17^ have shown that MNF/Foxk1, which mediates its DNA binding, recognizes the DNA sequence motif WRTAAAAYA and regulates the *c-myc*^16^ or *p21*^18^ gene. We recently found that FOXK1 was overexpressed in 16 cancerous human tissue types (including gastric cancer (GC)), induced tumor cell EMT, maintained the invasive potential of colorectal cancer and appeared to have a crucial role in the metastatic progression of human carcinomas.

C-jun might function as an oncogene by providing signals for cell survival, and is highly overexpressed in invasive human cancers.^19, 20, 21, 22^ Several studies have shown that c-jun sites are required for cell-specific gene expression. Virolle *et al.*^23^ demonstrated that DNA conformation, through the cooperative effect of c-jun binding sites, triggers keratinocyte-specific expression of the human *LAMA3* gene. Zutter *et al.*^24^ reported that two tandem c-jun sites with dyad symmetry are critical for megakaryocyte/platelet-specific enhancer activity. We identified a positive correlation between c-jun and FOXK1 expression patterns in GI cancers.^25^ However, the mechanism by which c-jun transcriptional activation regulates FOXK1 expression to promote cell growth, EMT, invasion and metastasis has not been investigated.

In this study, we showed that FOXK1 expression is elevated in cancer tissues and that FOXK1 overexpression promotes the proliferation, migration and invasion of GCs. Moreover, FOXK1 overexpression enhances EMT induction by TGF-*β* in MKN28 cells. Finally, c-jun regulates FOXK1 expression by transcriptional activation in human GC cells, thereby promoting cell growth, invasion and metastasis *in vitro* and *in vivo*. Our results support a potentially important role for FOXK1 in promoting human GC growth, invasion and metastasis.

## Results

### FOXK1 facilitates the malignant biological behavior of GC cells

**Figure 1:**
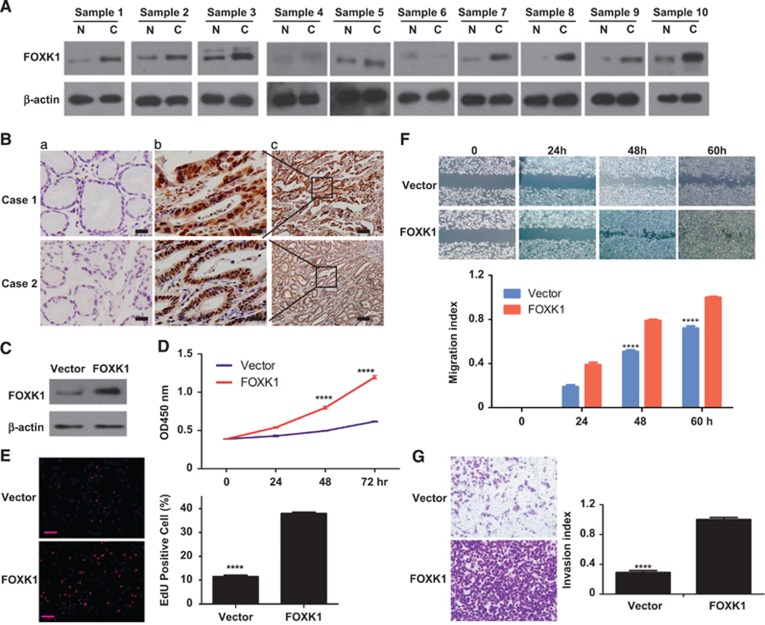
FOXK1 facilitates the malignant biological behavior of GC cells. (**A**) Proteins isolated from resected tumor and adjacent non-tumor tissue specimens were subjected to western blot analysis. T, GC tissues: N, normal tissues. *β*-Actin was used as the internal control. (**B**) FOXK1 expression in normal (a) or cancerous gastric tissue specimens (b and c) was detected using immunohistochemical (IHC) assays. (**C**) FOXK1 expression was detected using western blot analysis. (**D**) The proliferation of stable FOXK1 transfectants and vector was evaluated using a WST-8 assay. ****P<0.0001. (**E**) DNA synthesis of BCR823 cells was measured using an EdU (5-ethynyl-2′-deoxyuridine) incorporation assay after the indicated transfection at 48 h. ****P<0.0001, between FOXK1 and vector. (**F** and **G**) FOXK1 overexpression significantly promoted migration and invasive ability compared with vector. ****P<0.0001. All experiments were repeated three times with identical findings. Scale bars, 100 *μ*m in (**b** and **e**)

We first examined FOXK1 expression in 10 pairs of human GC tissues and matched non-cancerous gastric mucosa by western blot analysis. As shown in Figure 1A, the majority (8/10) of cancer tissues (C) exhibited higher FOXK1 expression level compared with the corresponding non-cancerous controls (N). The expression and subcellular localization of FOXK1 were determined using IHC analysis. Strong FOXK1-positive signals were present in the cancer cell nuclei (Figure 1Bb and c). Notably, positive signals were not found in the epithelial cells of normal gastric glands (Figure 1Ba).

To assess the effect of constitutive FOXK1 expression on the malignant behavior of GC cells *in vitro*, we established stable transfectants with FOXK1-sense and vector plasmids (as confirmed using western blot analysis; Figure 1C). The WST-1 and EdU incorporation assay revealed that cell proliferation were significantly higher compared with that in the control vector cells (Figures 1D and E). Similarly, the cell migration and invasion potential of FOXK1 cells were significantly higher compared with those of vector cells (Figures 1F and G).

These results indicate that FOXK1 facilitates the malignant biological behavior of GC cells.

### FOXK1 induces the TGF-*β*1-mediated EMT transition in human GC

**Figure 2:**
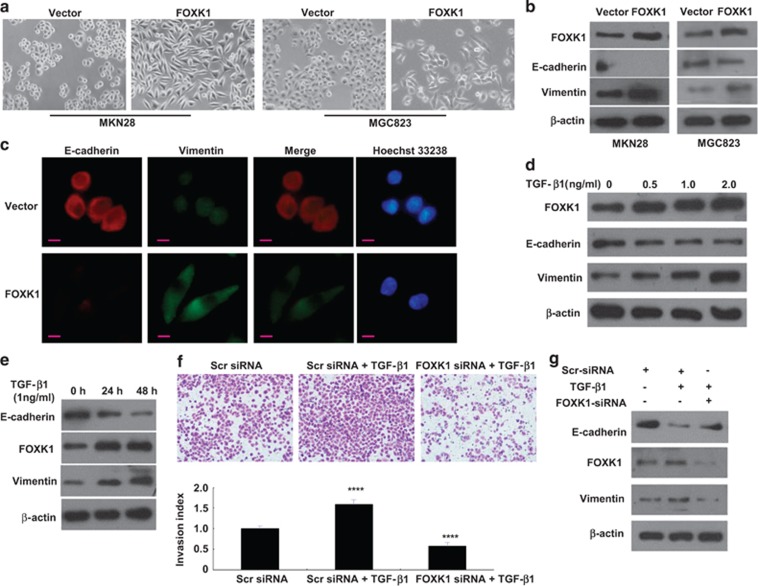
FOXK1 overexpression enhances TGF-*β*1-induced EMT. (**a**) Morphology of stable transfectants of the MKN28/vector and MKN28/FOXK1 as visualized using phase-contrast microscopy. (**b**) EMT biomarkers were detected using western blot analysis 48 h after transfection. (**c**) Immunofluorescence and microscopic visualization of E-cadherin (red) and vimentin (green) staining in FOXK1 and vector cells. (**d** and **e**) Western blot analysis of E-cadherin and vimentin with proteins extracted from MKN28 cells that were treated with various doses of TGF-*β* for various times. (**f**) Representative figures and data obtained from transwell assays for the indicated cells after treatment with TGF-*β*1. ****P<0.0001 between Scr small interfering RNA (siRNA) and Scr siRNA+TGF-*β*1; ****P<0.0001. FOXK1 siRNA+TGF-*β*1 and Scr siRNA+TGF-*β*1. (**g**) At 24 h after transfection with Src or FOXK1 siRNA, MKN28 cells were treated with TGF-*β* 1 (2 ng/ml) for an additional 48 h. FOXK1 expression was detected using western blot analysis. The results were reproduced in three independent experiments. Scale bars represent 20 *μ*m in (**a**) and 10 *μ*m in (**c**)

To further investigate the role of FOXK1 in EMT in GC, we examined the cell morphology. FOXK1 overexpression in the cells induced the loss of cell–cell contact and the cobblestone-like phenotype; the cells consequently became elongated, spindle shaped and scattered (Figure 2a). In contrast, empty vector transfectants displayed a round or flat morphology with a short cytoplasmic process. Upregulation of mesenchymal markers (vimentin) and downregulation of an epithelial marker (E-cadherin) were observed by western blot after the stable expression of FOXK1 (Figure 2b). E-cadherin was downregulated, as observed in the immunofluorescence analysis of MKN28/FOXK1 cells (Figure 2c).

EMT is reportedly induced by various signals in the tumor microenvironment, including TGF-*β*1.^26, 27^ We aimed to explore whether FOXK1 is involved in TGF-*β*1-induced EMT in GC cells. As expected, FOXK1 expression was increased in a time- and dose-dependent manner by TGF-*β*1 treatment (Figures 2d and e). This induction also increased the expression of a mesenchymal molecule, vimentin, while decreasing the expression of E-cadherin, an epithelial molecule (Figures 2d and e).

Furthermore, we identified the respective contribution of FOXK1 in TGF-*β*1-induced cell invasion. In a transwell assay, TGF-*β*1-induced cell motility was significantly abolished in FOXK1 siRNA-infected cells (Figure 2f). In contrast, TGF-*β*1 induced a greater increase in GC cell invasion compared with that in scrambled (Scr) siRNA. Consistently, FOXK1 knockdown altered the expression of EMT markers; E-cadherin expression was significantly upregulated, and vimentin expression evidently decreased (Figure 2g).

These data suggest that FOXK1 is involved in TGF-*β*1-induced EMT.

### FOXK1 is a direct transcriptional activation target of c-jun

**Figure 3:**
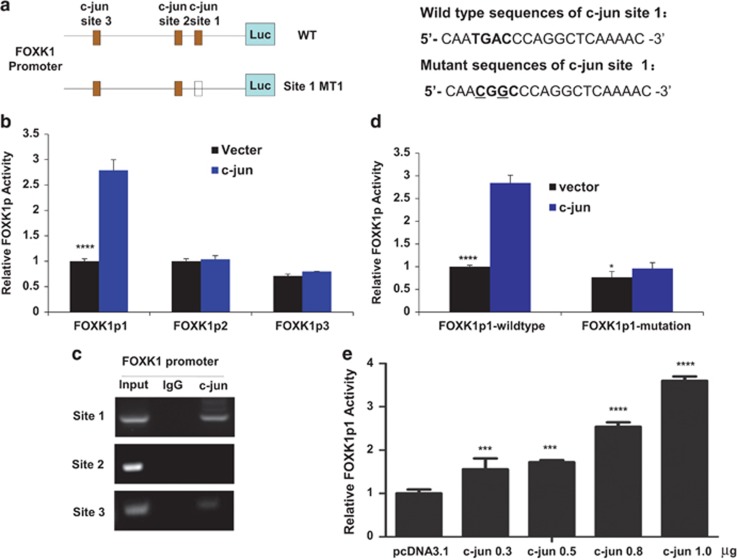
C-jun upregulates FOXK1 expression by directly binding to the proximal promoter of the *FOXK1* gene. (**a**) Schematic representation of the promoter region of FOXK1 (FOXK1p). The luciferase reporter constructs (FOXK1p1, FOXK1p2 and FOXK1p3) contained the FOXK1 promoter with three potential c-jun binding sites upstream of a luciferase gene. WT: wild type; MT: mutated. (**b**) The reporter constructs used in c-jun transfection experiments. C-jun activates the FOXK1 promoter after co-transfection in MKN28 cells. Luciferase activity was measured 48 h after transfection. Luciferase activity is expressed as the ratio of promoter reporter activity to control vector luciferase activity. ****P<0.0001. (**c**) ChIP was performed using an anti-c-jun antibody or control immunoglobulin G (IgG). The FOXK1 promoter region to which c-jun bound was significantly enriched after immunoprecipitation with an anti-c-jun antibody. (**d**) Primer sequences used to generate site-directed mutations. The FOXK1p1 (wild type 1) location was labeled in bold and mutated nucleotides are underlined. Site-directed mutagenesis of FOXK1p1 was performed to generate FOXK1p1-MT. ****P<0.0001; *P>0.05. (**e**) The c-jun protein stimulated FOXK1p1 activity, and the relative luciferase activity was enhanced by c-jun in a dose-dependent manner. ***P<0.001; ****P<0.0001

We analyzed the FOXK1 promoter using the Promo software (http://alggen.lsi.upc.es/cgi-bin/promo_v3/promo/promoinit.cgi?dirDB&equals;TF_8.3) and found several binding sequence transcription factors, including c-jun, HMAG1, Sp1, smad3 and smad4. We then investigated whether FOXK1 is directly regulated by c-jun. We surveyed FOXK1 in the proximal promoter (<600 bp) regions and identified the following three potential c-jun binding sites: site 1, −362 to −355; site 2, −386 to −379; and site 3, −571 to −564 (numbering is from the transcription start site; Figure 3a).

We then cloned the promoter regions c-jun-p362 (FOXK1p1), c-jun-386 (FOXK1p2) and c-jun-571 (FOXK1p3) of human FOXK1 upstream of a luciferase gene in a reporter plasmid. Transient transfections were performed to investigate whether the FOXK1 promoter was activated by c-jun overexpression. Dual-luciferase assay showed that the activity of FOXK1p1 in c-jun cells increased >3-fold compared with vector cells, whereas the magnification exhibited a slight decrease with FOXK1p2 and FOXK1p3 transfection (Figure 3b).

To confirm that c-jun could physically bind to the FOXK1 promoter *in vivo*, we performed chromatin immunoprecipitation (ChIP) assays in an MKN28 cell line expressing endogenous c-jun. The FOXK1 promoter region containing site 1 (c-jun-1) exhibited significant enrichment after immunoprecipitation with an anti-c-jun antibody. No bands were evident in immunoprecipitates obtained with the site 2 (c-jun-2) and site 3 (c-jun-3) possible binding site or control IgG (Figure 3c).

To verify the functional relationship between c-jun binding site 1 and FOXK1 promoter activity, mutation was introduced into FOXK1p1. Disruption of the FOXK1p1-mut1 by site-directed mutagenesis eliminated the transcriptional activity (Figure 3d).

We further characterized the FOXK1p1 plasmid of MKN28 cells. We showed that c-jun protein could stimulate FOXK1p1 activity, and the relative luciferase activity was enhanced by c-jun in a dose-dependent manner (Figure 3e).

Taken together, these data demonstrate that c-jun transactivated the FOXK1 promoter by binding to the c-jun-p362 region.

### FOXK1 and c-jun expression are positively correlated in GC

**Figure 4:**
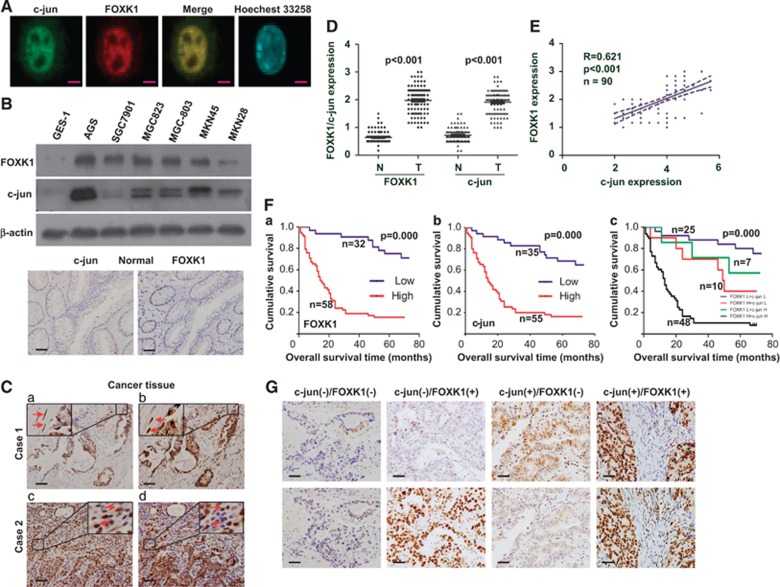
Positive correlation between c-jun and FOXK1 expression in GC. (**A**) Double staining of FOXK1 and c-jun in MKN28 cells in an indirect immunofluorescence assay; the nuclei were counterstained using Hoechst 33258. (**B**) FOXK1 and c-jun expression levels were detected in GC cell lines and in the immortalized normal gastric epithelial cell line GES-1 using western blot analysis. (**C**) C-jun (**a** and **c**) and FOXK1 (**b** and **d**) expression levels in normal or cancerous gastric tissue specimens were detected using immunohistochemical (IHC) assays. Normal mouse IgG was used as the isotype control for the first antibody (**a** and **b**). (**D**) The average scores of the two proteins in normal and cancerous GC tissues. *P*<0.001 between normal and cancer tissues. (**E**) FOXK1 and c-jun-positive staining was quantified, and the correlation between these proteins was analyzed using the Spearman's correlation method. *P*<0.001. (**F**) Kaplan–Meier overall survival analysis of GC patients. Survival analysis was performed according to the expression status of FOXK1 (**a**) and c-jun (**b**), as well as the combined expression status of FOXK1 and c-jun (**c**). (**G**) Representative IHC images for tissues are shown. Scale bars represent 20 *μ*m in (**A**) and 100 *μ*m in (**C** and **G**)

Because c-jun has previously been implicated in cancer cell growth and metastasis,^21, 22, 28^ we investigated whether c-jun and FOXK1 expression are correlated in GC. We first clarified the cellular distribution of the two proteins. A two-color immunofluorescence assay showed that the endogenous c-jun and FOXK1 proteins were localized to the nucleus of MKN28 cells. A merged signal indicated that the two proteins colocalized (Figure 4A). Second, we investigated the expression of these proteins in GC cell lines and in the immortalized normal gastric epithelial cell line GES-1 by western blot analysis. The FOXK1 expression pattern was very similar to that of c-jun in five of the above six cell lines, but not in SGC7901 (Figure 4B).

To validate our findings *in vivo*, we investigated the correlation between c-jun and FOXK1 expression in 90 pairs of adjacent normal gastric mucosal tissues and cancer tissues. Both proteins were highly expressed by cancer cells but were either not expressed or expressed at extremely low levels in normal tissues. Observation of serial sections showed that c-jun and FOXK1 were mainly located in the nucleus of cancer and tumor-associated stromal (TAS) cells (arrow), as illustrated in Figure 4C. Semiquantitative scoring showed that both proteins were expressed at significantly higher levels in cancer tissues compared with that in adjacent normal gastric mucosa tissues (Figure 4D). After calculating the regression coefficient between the expression scores of c-jun and FOXK1, we observed a significant correlation between c-jun and FOXK1 (*R*=0.621) in primary GC (Figure 4E).

These results might indicate that a strong positive correlation exists between FOXK1 and c-jun expressions in GC.

### Coexpression of FOXK1 and c-jun correlates with a poor prognosis in human GC

To explore the clinical relevance of FOXK1 and c-jun expression, we analyzed their clinicopathological features in GC. In a study of tumor samples obtained from 90 patients, FOXK1 and c-jun expression were significantly correlated with tumor differentiation, AJCC (American Joint Committee on Cancer) stage, lymph node metastasis and serosal invasion; however, the expression levels were not correlated with gender, age or tumor size (Supplementary Table 2).

To analyze the correlation between FOXK1 and c-jun expression with GC prognosis, Kaplan–Meier survival curves were generated. High positive expression of each protein was correlated with poor outcome (Figure 4Fa and b). Doubly positive cases (expressing both proteins) exhibited the worst prognosis (Figure 4Fc). Representative IHC images for tissues are shown in Figure 4g.

Univariate Cox regression model analysis revealed that poor survival was significantly associated with differentiation, AJCC stage, lymph node metastasis, serosal invasion, FOXK1 expression and c-jun expression (Supplementary Table 3). Based on the results of the univariate survival analysis, multivariate survival analysis was performed. After adjustment, tumor size, tumor differentiation, AJCC stage, FOXK1 expression and c-jun expression were identified as covariates. Serosal invasion and lymph node metastasis were excluded from the multivariate survival analysis because of their interactions with AJCC stage. AJCC stage and FOXK1 expression but not c-jun expression was considered independent risk predictors for poor overall survival (Supplementary Table 3).

The clinical sample results expanded our findings in model systems, showing that FOXK1 and c-jun are coexpressed and associated with metastasis and invasion, thereby influencing the GC prognosis.

### FOXK1 promotes GC development and progression by regulating c-jun

**Figure 5:**
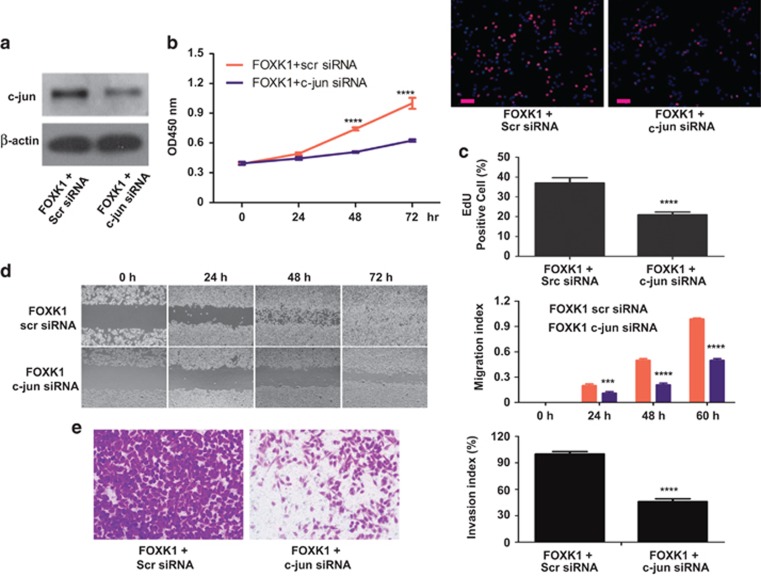
FOXK1 enhances c-jun-mediated participation in GC cell growth, migration and invasion. (**a**) C-jun expression levels were detected using western blot analysis in MKN28 cells, which were transfected with FOXK1 overexpression plasmids. This was followed by transfection with c-jun small interfering RNA (siRNA) or Scr siRNA as a negative control. (**b**) Stable transfectants of FOXK1 were then transfected with c-jun siRNA or Scr siRNA, cultured in complete medium for 24, 48 and 72 h and evaluated using a WST-8 assay. ****P<0.0001, between FOXK1-c-jun siRNA and FOXK1-Src siRNA. (**c**) MKN28-stable transfectants of FOXK1 after transfection with c-jun siRNA or Scr siRNA for 48 h were subjected to an EdU (5-ethynyl-2′-deoxyuridine) incorporation assay. ****P<0.0001. (**d**) For the wound-healing experiments, cells were analyzed using live-cell microscopy. Original magnification, × 10. ***P<0.001 and ****P<0.0001. (**e**) MKN28-stable FOXK1 transfectants were transfected with c-jun siRNA 48 h later, and the invasive ability of the cells was decreased. ****P<0.0001. Scale bars represent 100 *μ*m in (**c**)

To address directly whether the effects of FOXK1 in promoting GC cell proliferation can be attributed to its activation of c-jun, a rescue experiment was performed. C-jun was downregulated in FOXK1-overexpressing cells using siRNA, and this effect was confirmed using western blot analysis (Figure 5a). C-jun downregulation decreased the FOXK1-mediated proliferation of MKN28 cells as shown using a WST-1 assay (Figure 5b). Using an EdU incorporation assay, c-jun silencing was found to have a destructive effect on FOXK1-stimulated DNA synthesis (Figure 5c). We next investigated the functional roles of FOXK1 and c-jun in GC cell migration and invasion. C-jun knockdown in FOXK1-overexpressing cells led to a decrease in the migratory and invasion potentials of FOXK1-overexpressing cells *in vitro* (Figures 5d and e). These results suggest that the effects of c-jun in mediating the cell proliferation, migration and invasion of MKN28 cells were mediated by FOXK1.

### C-jun is required for FOXK1-mediated EMT ***phenotypes and metastatic*** potential *in vitro*

**Figure 6:**
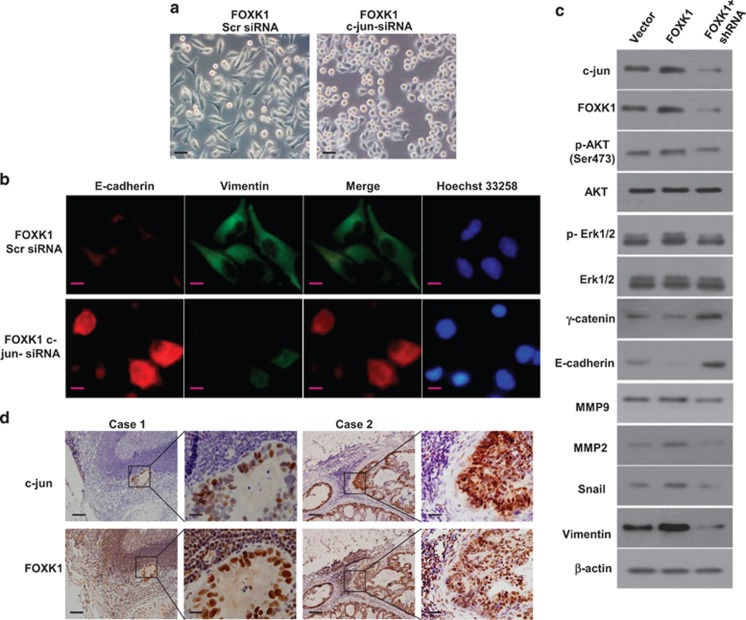
C-jun is critical for FOXK1-mediated EMT and metastatic phenotypes. (**a**) The aberrant morphology of stably expressing FOXK1 transfected with c-jun small interfering RNA (siRNA) or Src siRNA in MKN28 cells viewed under phase-contrast microscopy. (**b**) E-cadherin was upregulated, and vimentin was downregulated, as shown by immunofluorescence analysis after FOXK1-c-jun siRNA in MKN28 cells. (**c**) Decreased c-jun expression inhibited the AKT/ERK/EMT signaling pathway, as detected using western blot analysis. (**d**) Representative immunohistochemical (IHC) images are shown for FOXK1 and c-jun expression in lymph node metastatic cancer tissues. Scale bars, 20 *μ*m in (**a**), 10 *μ*m in (**b**) and 100 *μ*m in (**d**)

To investigate whether FOXK1 regulates c-jun expression, we examined the morphologic features of these cells. FOXK1 transfectants exhibited a spindle-like, fibroblastic morphology. Long or dendritic-like cytoplasmic processes were visible under a phase-contrast microscope. C-jun knockdown in FOXK1-overexpressing MKN28 cells led to EMT reversion (Figure 6a). Immunofluorescence staining of E-cadherin and vimentin confirmed the EMT-associated shift in marker expression (Figure 6b). Next, we performed western blot analysis to elucidate the phosphorylation status of proteins that are involved in EMT signaling. The phosphorylation levels of AKT and ERK1/2 were downregulated after c-jun knockdown in FOXK1-overexpressing cells compared with FOXK-overexpressing cells, and the total amount of AKT and ERK1/2 protein was unaltered. Moreover, the expression levels of the typical EMT epithelial markers E-cadherin and *γ*-catenin were upregulated after c-jun knockdown in FOXK1-overexpressing cells. In contrast, the mesenchymal markers MMP-2, MMP-9, vimentin and Snail were downregulated (Figure 6c).

We detected FOXK1 and c-jun expression in regional lymph node metastatic GC tissue and found that FOXK1 and c-jun were expressed at high levels in the nucleus of cancer cells (Figure 6d). This result confirmed the positive correlation between FOXK1 and c-jun that was observed using IHC.

Taken together, these data suggest that the FOXK1-c-jun axis promotes the invasion and metastasis of GC cells.

### FOXK1 is essential for the induction of tumorigenesis and metastasis by c-jun *in vivo*

**Figure 7:**
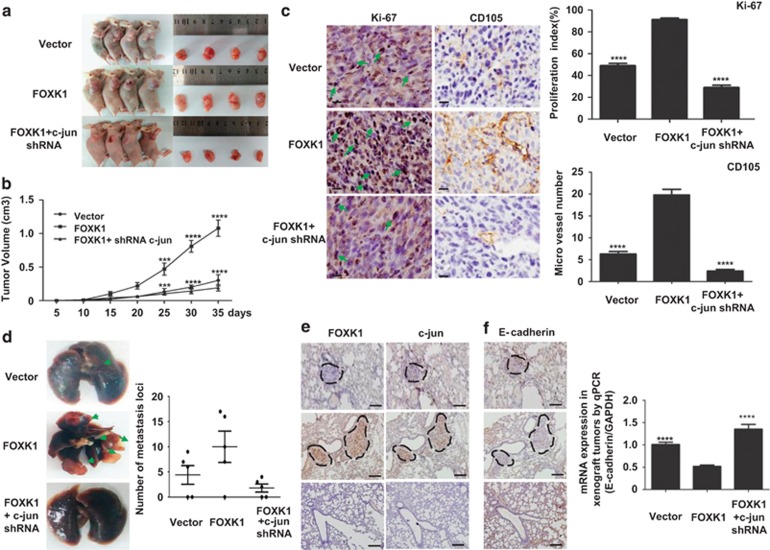
C-jun facilitates FOXK1-mediated cell proliferation, EMT and liver metastasis in GC *in vivo*. (**a**) Evaluation of tumorigenesis in nude mice that were subcutaneously injected with MKN28-vector, MKN28-FOXK1 and MKN28-FOXK1-c-jun short hairpin RNA (shRNA) cells. The images were captured on day 35 after injection. (**b**) Tumor size was measured weekly after tumor cell inoculation in each group. ***P<0.001; ****P<0.0001; vector *versus* FOXK1 and FOXK1 *versus* FOXK1-c-jun shRNA, respectively. (**c**) C-jun knockdown significantly inhibited FOXK1-induced proliferation (Ki-67, ****P<0.0001, vector *versus* FOXK1 and FOXK1 *versus* FOXK1-c-jun shRNA, respectively), and a considerable decrease of tumor vessel density (CD105, ****P<0.0001, vector *versus* FOXK1 and FOXK1 *versus* FOXK1-c-jun shRNA) was observed using immunohistochemisty (IHC). (**d**) Mice were orthotopically transplanted with MKN28 cells (*n*=3 in each group). Representative images of metastatic loci in the lungs are shown. The number of metastatic loci in the lung was counted (right). (**e**) Confirmation of FOXK1 and c-jun expression by IHC staining. (**f**) The expression of E-cadherin in tumors derived from MKN28 cells was determined using IHC staining quantitative PCR (qPCR). ****P<0.0001; vector *versus* FOXK1; FOXK1 *versus* FOXK1-c-jun siRNA. Scale bars represent 100 *μ*m in (**c**) and 200 *μ*m in (**d**). siRNA, small interfering RNA

To determine whether FOXK1 regulates c-jun expression *in vivo*, we injected MKN28 cells subcutaneously into the right flank of nude nu/nu mice. As shown in Figures 7a and b, xenografted FOXK1-overexpressing cells rapidly proliferated in mice compared with vector-expressing cells. However, xenografted tumor growth was suppressed in mice that were injected with FOXK1-overexpressing cells in which c-jun was downregulated compared with FOXK1-overexpressing cells with normal c-jun levels.

We next examined the protein expression of cell proliferation (Ki-67) and angiogenesis (CD105) markers in the xenograft tumors. Representative images of the tumors after IHC staining are shown in Figure 7c. The FOXK1-stable transfectant group exhibited a significantly increased proliferation rate and tumor vessel density compared with the vector group, whereas c-jun knockdown inhibited the growth rate and tumor vessel density in the FOXK1-overexpressing group.

To test the role of c-jun in FOXK1-mediated tumor progression, we injected cells into nude mice via the tail vein, which results in lung metastasis within 40 days. FOXK1-overexpressing MKN28 cells, but not control MKN28 cells, formed a variety of large metastatic nodules in the lung. Compared with c-jun downregulation in FOXK1-overexpressing cells, FOXK1-overexpressing cells exhibited a significant increase in visible lung tumors, and this was correlated with a higher number of metastasis loci (Figure 7d).

To further determine whether c-jun is required for FOXK1-mediated EMT, c-jun repression in FOXK1-overexpressed cells was performed using IHC and real-time PCR (RT-PCR) in orthotopic xenograft tumors. IHC staining confirmed FOXK1 and c-jun expression in the xenografted tumors (Figure 7e). Moreover, the overexpression of FOXK1 resulted in a significant loss of E-cadherin, whereas downregulation of c-jun in FOXK1-overexpressing cells caused an increase in E-cadherin (Figure 7f). Thus, we suggested that the critical role of c-jun in the induction of EMT might require FOXK1 expression.

It is suggested that the critical role of c-jun in the induction of EMT is caused by expression of FOXK1.

## Discussion

In this study, we characterized the role of FOXK1 in GC cell growth, invasion and metastasis. We found that FOXK1 was overexpressed in cancer tissues and further identified, for the first time, the role of FOXK1 in promoting EMT. Moreover, we identified the functional region of the FOXK1 promoter and found that c-jun bound to and regulated the gene expression of FOXK1. Our results indicate that c-jun has a critical role in FOXK1-mediated tumor growth, EMT and metastatic phenotypes *in vitro* and *in vivo*.

Previous evidence had indicated that FOXK1 acts as a tumor oncogene in colorectal cancer. Wang *et al.*^29^ found that FOXK1 and FOXK2 positively regulate Wnt/*β*-catenin signaling by translocating DVL into the nucleus. However, the role of FOXK1 in GC remains unclear. We found that FOXK1 was overexpressed in GC cells and tissues. Moreover, FOXK1 overexpression induced EMT, causing a loss of epithelial polarity. A stable transfectant of FOXK1 promoted migration, metastasis and dissemination, thereby facilitating tumor development and progression in GC cells. These findings imply that aberrant FOXK1 upregulation might be an important mechanism underlying cancer metastasis.

The TGF-*β* signaling pathway has pivotal roles in diverse developmental processes and the pathogenesis of many diseases, including cancer. TGF-*β* activates a membrane receptor serine/threonine kinase complex that phosphorylates the transcription factors Smad2 and Smad3. Thus, activated, Smad2/3 accumulates in the nucleus and bind Smad4, which is essential for many, but not all, Smad-dependent responses.^30, 31^ A key feature of TGF-*β* signaling activation is that the SMAD2 or SMAD3 proteins in activated SMAD4-SMAD2/SMAD3 complexes in the nucleus bind other DNA-binding transcription factors as partners for target gene recognition and transcriptional regulation. For example, Luo *et al.*^32^ characterized that Ski can interact directly with Smad2, Smad3 and Smad4 on a TGF-*β*-responsive promoter element. In this study, we characterized FOXK1 as a TGF-*β*-responsive gene. We analyzed the FOXK1 promoter using the Promo software and found several binding sequence transcription factors, including c-jun, smad3 and smad4. Our results suggest that FOXK1 may be an upstream target of TGF*-β/*Smad signaling pathway.

EMT is a differentiation process of epithelial cells into mesenchymals, which is widespread in the invasion and metastasis of malignant tumor. TGF-*β*1 can induce EMT in the majority of epithelial cell type tested and in transgenic mouse. Recently, some members of the Forkhead factor family have been shown to have a role in mediating TGF-*β*-induced EMT. FOXC2^33^ was overexpressed in invasive ovarian cancer cell lines and tissues. Moreover, FOXC2 was required for maintaining the mesenchymal phenotype after TGF-*β*1-induced EMT in human ovarian cancer cells. FOXQ1^34^ promoted invasion and metastasis in colorectal cancer cells that had undergone TGF-*β*-induced EMT. Among the forkhead proteins, we found that FOXK1 has an important role in EMT, and TGF-*β*1 has been identified as the most potent factor. TGF-*β*1 induced morphological changes, concomitantly decreased E-cadherin expression and increased vimentin expression, and promoted GC cell invasiveness. In contrast, FOXK1 knockdown slowed TGF-*β*-induced EMT. Our findings suggest that FOXK1 acts as a costimulator in TGF-*β*1-induced EMT in GC.

The c-jun proto-oncogene encodes the founding member of the AP-1 family.^21, 22, 35^ C-jun is overexpressed in many human cancers and has been shown to be associated with a diverse range of important cancer characteristics, including cell proliferation,^36^ invasive capacity,^37^ angiogenesis^38^ and drug resistance.^39^ Disruption of the *c-jun* gene in murine hepatocytes prevents the emergence of hepatocellular carcinoma,^40^ and c-jun is sufficient for inducing the anchorage-independent growth of Rat1a cells.^41^ We analyzed the FOXK1 promoter using the Promo software. The FOXK1 proximal promoter harbored three c-jun binding sites, which consist of the nucleotide sequence 5′-TGACTTG-3′. In the present study, we found that c-jun expression is involved in FOXK1 promoter activity in GC cells, which we identified using a ChIP assay and a luciferase reporter system. The sequence from −362 to −355 was identified as a c-jun 1 binding site. Mutations of the active site profoundly attenuated the c-jun-mediated transactivation of FOXK1 promoters. Thus, we identified that FOXK1 is a direct transcriptional target of c-jun.

We observed that the distribution pattern of FOXK1 is highly congruent with that of c-jun and that FOXK1 protein expression is highly correlated with c-jun expression using immunohistochemistry and immunofluorescence. Moreover, linear correlation between FOXK1 and c-jun expression was observed in GC. Further survival analysis indicated that the overexpression of FOXK1 and/or c-jun predicted a poor prognosis. Thus, our study further confirmed that FOXK1 and/or c-jun overexpression can be considered an unfavorable prognostic biomarker for patients with CRC.

Smith *et al.*^42^ previously demonstrated that c-jun has a critical role in the migration and invasion characteristics of a human breast cancer cell line *in vitro*. We further investigated whether c-jun contributes to FOXK1-induced EMT and metastasis. We silenced c-jun expression in MKN28 cells that were transfected with a FOXK1-expressing plasmid and found that the EMT phenotype was reversed. Moreover, c-jun knockdown inhibited EMT and the metastatic potential induced by FOXK1 overexpression in GC *in vitro* and *in vivo*. These results strongly suggest that the induction of the EMT and metastasis by c-jun is mediated by FOXK1.

In conclusion, FOXK1 is markedly overexpressed in GC and enhances tumorigenicity and tumor growth *in vivo*. Moreover, we identified FOXK1 as a direct target of c-jun in GC. FOXK1/c-jun promotes the proliferation, migration and invasion, and metastasis of GC cells and might represent a novel target for treating GC.

## Materials and Methods

### Reagents, cells and culture conditions

See Supplementary Information.

### Patients and specimens

The FOXK1 and c-jun staining results were classified according to the carcinoma cell staining intensity as follows: 0, negative staining; 1, weak staining; 2, moderate staining; and 3, intense staining. We defined negative- and weak-stained cells as low expressers, and cells that were moderately and intensely stained were considered to be high expressers of this protein. The average score for each sample evaluated by two observers was considered as the final IHC score.

### Plasmid

See Supplementary Information.

### EdU incorporation and WST-1 cell proliferation assay

See Supplementary Information.

### Western blot assays and immunofluorescence

For western blot analysis, cells were harvested and lysed in lysis buffer. A total of 30 *μ*g of protein lysates were separated by SDS-PAGE and transferred onto a PVDF membrane. Primary antibodies were diluted according to the company's recommendation. Protein bands were detected by using an ECL Western Blotting Detection Reagent (Merck Millipore, Darmstadt, Germany).

Cells grown in cover glass were fixed with 4% paraformaldehyde, and the non-specific bindings were block by incubation with 3% BSA. The glasses were probed with the first antibodies followed by TR- (Texas red) or FITC-conjugated second antibodies. Nuclei were counterstained with 1 *μ*g/ml Hoechst 22358 and sealed with nail varnish. Confocal images were captured with a Zeiss LSM710 confocal microscope using the 40x objectives.

### Invasion and cell migration assays

Invasion and cell migration assays were performed as described.^26^ Cells were plated in serum-free medium on Transwell inserts (Corning, NY, USA) coated with 25 *μ*g of Matrigel (BD Biosciences, Piscataway, NJ, USA) for invasion assays. After incubation for 48 h at 37 °C/5% CO_2_, the inserts were fixed with 3.7% paraformaldehyde/PBS and stained with 2% crystal violet. The number of cells that had invaded was counted in five representative (× 200) fields per insert. Cell migration assays were performed. Briefly, cells plated in six-well plates with 100% confluence were wounded with a pipette tip at time 0. Media were changed to remove cell debris and the cells were cultured in the presence of 10 *μ*g/ml mitomycin C to inhibit cell proliferation. Photographs were taken every 12 h.

### Quantitation of mRNA by reverse transcription-PCR

See Supplementary Information.

### Promoter reporter and dual-luciferase assays

First, 362-bp (FOXK1p1), 386-bp (FOXK1p2) and 571-bp (FOXK1p3) fragments of the FOXK1 promoter upstream of the transcription start site were cloned into the pGL3basic vector. For the luciferase assay, the cells were transiently transfected with the various pLuc constructs with Lipofectamine 2000 (Invitrogen, Carlsbad, CA, USA). The firefly and *Renilla* luciferase activities were measured using the Dual-Luciferase reporter system (Promega, Madison, WI, USA) with a model TD-20/20 Luminometer (Turner Designs, San Jose, CA, USA). The firefly luciferase activity value was normalized to the *Renilla* activity value. The transcriptional activity at the promoter was presented as the fold induction of relative luciferase units (RLUs) compared with the basic pGL3 vector control. The RLU was the value of the firefly luciferase unit divided by the value of the *Renilla* luciferase unit. All treatments were triplicated for each single experiment. Site-directed mutagenesis of potential c-jun binding sites was carried out in the FOXK1p1 and FOXK1p2 plasmid using the ClonExpress II One Step Cloning Kit (Vazyme, Nanjing, China). All mutations were verified by sequencing. The primer sequences are listed in Supplementary Table 1.

### ChIP assays

See Supplementary Information.

### Gene silencing using siRNA

c-jun siRNA and Scr control siRNA were purchased from Genepharma Company (Suzhou, China). The lentivirus which contain c-jun shRNA and control shRNA respectively were purchased from Genechem Company (Shanghai, China). Cells were transfected using Lipofectamine 2000 for 4 h following which the lipid and siRNA complex was removed and fresh growth medium was added. Cells were lysed 48 h after transfection and specific protein levels were determined by western blot analysis with specific antibodies.

### Construction and transfection of lentiviral vectors

See Supplementary Information.

### *In vivo* assays for tumor growth and metastasis

MKN28 cells (5 × 10^6^) that had been transfected with pcDNA3.1, pcDNA3.1-FOXK1 and pcDNA3.1-FOXK1-c-jun siRNA lentivirus were suspended in 100 *μ*l serum-free RPMI, implanted subcutaneously into the flanks of nude mice (four in each group, female BALB/c nu/nu, 4–6 weeks old; Laboratory Animal Unit, Southern Medical University, Guangzhou, China). The resulting tumor sizes were measured weekly. The tumor volumes were calculated as follows: total tumor volume (mm^3^)=*L* × *W*^2^/2, where *L* is the length and *W* is the width. On day 35 after inoculation, the mice were killed, and the tumors were dissected and weighed.

The proliferation index was determined by Ki-67 immunostaining and calculating the ratio of Ki-67-positive cells among total number of cells in five randomly selected fields at × 200. The sections were stained with anti-mouse CD105 monoclonal antibody (BD Pharmingen, San Diego, CA, USA). Immunohistochemical assessment of tumor microvessels in five randomly selected fields at × 200. The average of microvessels number was calculated.

To evaluate the *in vivo* metastatic potential of cancer cells, the mice were injected into 4 × 10^5^/pcDNA3.1, pcDNA3.1-FOXK1 and pcDNA3.1-FOXK1-c-jun shRNA lentivirus cells per mouse through tail vein. After 40 days, the mice were killed and the lungs were harvested and photographed. Tissue sections were attained with the traditional method and HE and IHC staining was performed.

### Statistical analysis

Statistical analysis was performed using the SPSS statistical software package (standard version 13.0; SPSS, Chicago, IL, USA). Quantitative data we got from experiments with biological replicates were shown as means (±S.D. or S.E.M). The mean from each group was compared using *t-*tests or one-way ANOVA. Linear regression and Pearson's correlation analysis were performed to assess the relationship between FOXK1 and c-jun in the tissue. Survival analysis were performed via Kaplan–Meier and log-rank test. Cox proportional hazards regression was performed to identify independent factors with a significant impact on patient survival. Probability values from the two-tailed test <0.05 were considered significant.^43^

## Abbreviations

EMT: epithelial-to-mesenchymal transition
TGF: transforming growth factor
RLU: relative luciferase unit
AJCC: American Joint Committee on Cancer
ChIP: chromatin immunoprecipitation
GC: gastric cancer
