# Supplementary material for: Direct regulation of FOXK1 by C-jun promotes proliferation, invasion and metastasis in gastric cancer cells
Source: Cell Death Dis. 2016 Nov 24;7(11):e2480–. doi: 10.1038/cddis.2016.225 (PMC5260906; doi:10.1038/cddis.2016.225)
Supplement: Supplementary Tables [file cddis2016225x1.docx]

**Supplementary Table 1. Primary Primers Used in This Study**

| Experiment | Name | Position or  orientation | Sequence (5’-3’) |
| --- | --- | --- | --- |
| Luciferase  construction | R: | -79 ~ -58 | TCCGCTCGAG GCTGTCCTCGCCGACTTC (Xho I) |
|  | L: FOXK1p1-WT | -365~ -345 | F: 5’ - GGGGTACC CAATGACCCAGGCTCAAAAC (Kpn I) -3’ |
|  | L:  FOXK1p2-WT | -388 ~ -368 | F: 5’- GGGGTACC CTGAGGTCATCCGTCCCTTT (Kpn I) -3’ |
|  | L:FOXK1p3-WT | -585 ~ -564 | F: 5’- CAGAGGCAGGAGGGTGACTTG (Kpn I) -3’ |
| Site-  directed mutagenesis | L:  FOXK1p1-MT | -366 ~ --335 | F: CCAATAATCCAGGCTCAAAACTTTGTGGCTG |
|  |  |  | R:  CAGCCACAAAGTTTTGAGCCTGGATTATTGG |
|  | L: FOXK1p2-MT | -399 ~ -364 | F:  CGGGGAGACTGCTGAGGCCGTCCGTCCCTTTCTCC |
|  |  |  | R:  GGAGAAAGGGACGGACGGCCTCAGCAGTCTCCCCG |
| Chip | Chip 1 | L: -377 ~ -357 | CGTCCCTTTCTCCAATGACC |
|  |  | R: -185 ~ -165 | TAGCCCACGTGGCGCGCGCC |
|  | Chip 2 | L:-557 ~ -377 | GAGGTGGAAGCTGCAGTGA |
|  |  | R:-383 ~ -364 | GGAGAAAGGGACGGATGAC |
|  | Chip 3 | L:-725 ~ -705 | CGGGTAATCCCAGTGCTTT |
|  |  | R:-506 ~ -486 | ATTCTAACCTCGCTCTGTCG |

**Supplementary Table 2. Correlation between FOXK1/c-jun protein expression and the clinicopathological parameters** of GC

| Features | Expression of FOXK1 | | | Expression of c-jun | | |
| --- | --- | --- | --- | --- | --- | --- |
|  | Low expreession(%) | High expression(%) | p | Low expreession(%) | High expression(%) | p |
| Age(years) | | | | | | |
| <60 | 12(31.6) | 26(68.4) | 0.500 | 13(34.2) | 25(65.8) | 0.436 |
| ≥60 | 20(38.5) | 32(61.5) |  | 22(42.3) | 30(57.7) |  |
| Gender | | | | | | |
| Male | 21(39.6) | 32(60.4) | 0.335 | 21(39.6) | 32(60.4) | 0.864 |
| Female | 11(29.7) | 26(70.3) |  | 14(37.8) | 23(62.2) |  |
| Differentiation | | | | | | |
| Well | 17(80.9) | 4(19.1) | 0.001 | 10(85.7) | 3(14.3) | 0.001 |
| Moderate | 12 (63.1) | 7( 36.9) |  | 14(73.6) | 9(26.3) |  |
| Poor | 3(6) | 47(94) |  | 11(14) | 43(86) |  |
| AJCC stage | | | | | | |
| I,II | 26(72.2) | 10( 27.8) | 0.001 | 28(77.7) | 8(22.3) | 0.001 |
| III,IV | 6(11.1) | 48(88.9) |  | 7(12.9) | 47(87.1) |  |
| Lymph node metastasis | | | | | | |
| No | 17(73.9) | 6(26.1) | 0.001 | 17(73.9) | 6(26.1) | 0.001 |
| Yes | 15(22.4) | 52(77.4) |  | 18(26.8) | 49(73.2) |  |
| Tumor size | | | | | | |
| <5cm | 24(42.9) | 32(57.1) | 0.063 | 25(44.6) | 31(55.3) | 0.151 |
| ≥5cm | 8(23.5) | 26(76.5) |  | 10(29.4) | 24(70.6) |  |
| Serosel invasion | | | | | | |
| Yes | 23(30.3) | 53(69.7) | 0.015 | 23(30.3) | 53(69.7) | 0.001 |
| No | 9(64.3) | 5(35.7) |  | 12(85.7) | 2(14.3) |  |

**Supplementary Table 3. Univariate and multivariate analyses of different prognostic factors in 90 patients with GC**

| Variable | All case | Univariate analysis | | Multivariate analysis | |
| --- | --- | --- | --- | --- | --- |
|  |  | HR (95% CI) | P-value | HR (95% CI) | P-value |
| Age (years) | | 0.990(0.969-1.011) | 0.343 |  |  |
| <60 | 38 |  |  |  |  |
| ≥60 | 52 |  |  |  |  |
| Gender | | 1.452（0.866-2.434） | 0.157 |  |  |
| Male | 53 |  |  |  |  |
| Female | 37 |  |  |  |  |
| Differentiation | | 1.949（1.276-2.976） | 0.002 | 0.862(0.534-1.392) | 0.544 |
| Well | 21 |  |  |  |  |
| Moderate | 19 |  |  |  |  |
| Poor | 50 |  |  |  |  |
| AJCC stage | | 8.972（4.287-18.775） | <0.001 | 4.770(2.046-11.117) | <0.001 |
| I,II | 36 |  |  |  |  |
| III,IV | 54 |  |  |  |  |
| Lymph node metastasis | | 8.643（3.108-24.038） | <0.001 |  |  |
| No | 23 |  |  |  |  |
| Yes | 67 |  |  |  |  |
| Tumor size | | 1.004（1.000-1.008） | 0.051 | 1.000(0.995-1.005) | 0.980 |
| <5cm | 56 |  |  |  |  |
| ≥5cm | 34 |  |  |  |  |
| Serosel invasion | | 3.937（1.422-10.902） | 0.008 |  |  |
| Yes | 76 |  |  |  |  |
| No | 14 |  |  |  |  |
| FOXK1 expression | | 6.188（2.997-12.78） | <0.001 | 2.883(1.224-6.792) | 0.015 |
| Low expression | 32 |  |  |  |  |
| High expression | 58 |  |  |  |  |
| c-jun expression | | 4.957(2.589-9.489) | < 0.001 | 1.746(0.797-3.822) | 0.163 |
| Low expression | 35 |  |  |  |  |
| High expression | 55 |  |  |  |  |
